# Prediction of Inhibitory Activity of Epidermal Growth Factor Receptor Inhibitors Using Grid Search-Projection Pursuit Regression Method

**DOI:** 10.1371/journal.pone.0022367

**Published:** 2011-07-21

**Authors:** Hongying Du, Zhide Hu, Andrea Bazzoli, Yang Zhang

**Affiliations:** 1 Center for Computational Medicine and Bioinformatics, University of Michigan, Ann Arbor, Michigan, United States of America; 2 Department of Public Health, Lanzhou University, Lanzhou, China; 3 Department of Chemistry, Lanzhou University, Lanzhou, China; University of Georgia, United States of America

## Abstract

The epidermal growth factor receptor (EGFR) protein tyrosine kinase (PTK) is an important protein target for anti-tumor drug discovery. To identify potential EGFR inhibitors, we conducted a quantitative structure–activity relationship (QSAR) study on the inhibitory activity of a series of quinazoline derivatives against EGFR tyrosine kinase. Two 2D-QSAR models were developed based on the best multi-linear regression (BMLR) and grid-search assisted projection pursuit regression (GS-PPR) methods. The results demonstrate that the inhibitory activity of quinazoline derivatives is strongly correlated with their polarizability, activation energy, mass distribution, connectivity, and branching information. Although the present investigation focused on EGFR, the approach provides a general avenue in the structure-based drug development of different protein receptor inhibitors.

## Introduction

The epidermal growth factor receptor (EGFR) is a transmembrane glycoprotein belonging to the human epidermal receptor (HER) family [1]. It is a type I tyrosine kinase receptor which plays a vital role in signal transduction pathways, regulating key cellular functions such as cell proliferation, survival, adhesion, migration, and differentiation [2]–[4]. The binding of a ligand to EGFR induces conformational changes within the receptor which increase its intrinsic catalytic activity of a tyrosine kinase and result in autophosphorylation, which is necessary for biological activity [5]–[7]. Mutations that lead to EGFR overexpression or overactivity have been associated with a variety of human tumors, including lung, bladder, colon, brain, and neck tumors [8]–[11]. Therefore, inhibitors of EGFR — inhibiting EGFR's kinase activity by competing with its cognate ligands — may potentially constitute a new class of effective drugs in clinical use or cancer therapy [12]–[14].

There are presently two main classes of EGFR inhibitors that can be used in cancer therapy. Both classes — the quinazoline derivatives [15]–[17] and the pyrimidin derivatives [18]–[20] — consist of ATP-competitive small molecules. To discover new effective EGFR inhibitors, investigators usually need to synthesize many compounds and test their corresponding activities by cell-based biological assay experiments, which is usually time-consuming and manpower expensive [21], [22]. Consequently, it is of practical interest to develop reliable tools to predict biological activities before synthesis.

Quantitative structure–activity relationship (QSAR) is the most popular theoretical method for modeling a compound's biological activity from its chemical structure [23]–[28]. Using this approach, scientists could predict the activities of series of newly designed drugs before making the final decision on whether or not to synthesize and assay them. The prediction is based on the structural descriptors of the molecular features that most account for the variations in biological activity. Furthermore, this method also can identify and describe the most important structural features of the compounds which are relevant to the variations in molecular properties, thus, it also gains an insight into the structural factors which affect the molecular properties. QSAR models of EGFR inhibitors have been recently investigated with encouraging results [29]–[33]. However, it is still vital to find faster and more reliable methods to assess the capability of EGFR inhibitors.

The exceedingly high dimension of the space of descriptors is a major problem in developing QSAR models. For this reason, increasing attention in the past several years has been devoted to QSAR models developed by projection pursuit regression (PPR) [34], [35]. This is a general statistical technique that seeks the “interesting” projections of data from high-dimensional to lower-dimensional space, with the purpose of extracting the intrinsic structural information hidden in the high-dimensional data [36].

In the current investigation, two QSAR models were constructed from a set of known quinazoline-derivative EGFR inhibitors using multi-linear and non-linear regression approaches. The stability and accuracy of the regression models were assessed through an independent test set of EGFR inhibitors and a 5-fold cross validation approach. The study sheds light on the structure–activity relationship of this class of EGFR inhibitors and has the potential prediction ability to identify new EGFR inhibitors. In addition, the explored structural features of the chemicals described here may facilitate the design of further new inhibitors with high pIC_50_ activities without any biological assay. Since the prediction relies exclusively on structural descriptors, the approach is expected to be of general use in drug design and discovery research.

## Materials and Methods

### Data set

The present investigation considered 128 quinazoline derivatives with known anti-cancer EGFR inhibitory activities [20], [30], [37]–[41]. The structures and activities of these compounds are listed in Table S1. The activities are expressed as pIC_50_ ( = −log (IC_50_)) values, where IC_50_ (nM) represents the concentration of these compounds that produces 50% inhibition of the kinase activity of EGFR. Our aim was to exploit these known experimental activities to develop a QSAR model that would predict, based on selected chemo-physical molecular descriptors, the EGFR inhibitory activity of potential hits from the virtual screening of a compound library. To this purpose, the set of known EGFR inhibitors was randomly divided into two subsets: a training set of 103 compounds and a test set of 25 compounds (marked by asterisks in Table S1). The training set served to construct the QSAR models, while the test set was used for the model validation.

### Generation of the molecular descriptors

**Figure 1:**
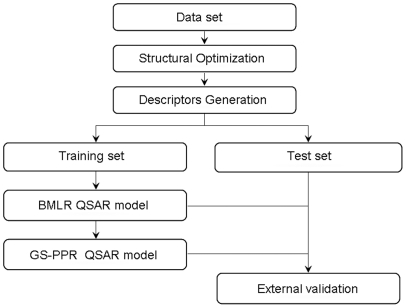
Flowchart of the QSAR study.

Two-dimensional structures of the compounds were drawn by using ISIS Draw 2.3 [42]. All the structures were fed into HyperChem 7.0 [43] and pre-optimized with the MM+ molecular-mechanics force field. The structures were then minimized in energy with the more precise semi-empirical AM1 method in MOPAC. After these steps, the DRAGON 5.4 [44] and CODESSA [45] programs were used to calculate the molecular descriptors from the structures, including 0D, 1D, 2D, and 3D descriptors from DRAGON [46], and constitutional, topological, geometrical, electrostatic, and quantum-chemical descriptors from CODESSA. 0D descriptors contain constitutional descriptors; 1D descriptors include functional-group counts and atom-centered fragments; 2D descriptors contain topological descriptors, connectivity indices, information indices, and eigenvalue-based indices; 3D descriptors represent some novel exclusive DRAGON descriptors and geometrical descriptors. There remained a total of 982 molecular descriptors after eliminating the constant and the highly-correlated descriptors. Then the generated descriptors were used to construct the regression models to predict the activities of the compounds. In order to clarify the whole procedure, its flowchart was drawn in Fig. 1.

### Best multi-linear regression

Best multi-linear regression (BMLR) refers to a multi-linear method which utilizes a more thorough procedure for finding the best linear regression model, based on a dependent variable and one or more independent variables [47]. The BMLR approach has several advantages, including high speed and no restriction on the size of the data set. It can either give a good estimation of the degree of correlation to expect from the data, or derive several best regression models. Moreover, it can highlight which descriptors have bad or missing values, which are insignificant, and which are highly inter-correlated. For these reasons, the BMLR method was chosen in this study to pre-select the most important descriptors and to construct the linear QSAR model.

### Projection pursuit regression

Projection pursuit regression (PPR), developed by Friedman and Stuetzle [34], is a powerful method for seeking the interesting projections from high-dimensional spaces into lower-dimensional ones by means of linear projections. It can overcome the curse of dimensionality because it relies on estimation at most trivariate settings. At present, it has been successfully applied to tackle several chemical problems [36], [48]. Friedman and Stuetzle's concept of PPR avoided many difficulties compared with other existing non-parametric regression procedures. Different from recursive partitioning regression, it does not split the predictor space into two regions, thereby allowing, when necessary, more complex models. In addition, interactions of predictor variables are directly considered because linear combinations of the predictors are modeled with general smooth functions. The basic theory of PPR can be found in references [34], [35]. Here, only a brief description is given. Let *X* be a (*k*×*n*) data matrix, where *k* is the number of observed variables and *n* is the number of units. Let also *A* be an *m*-dimensional orthonormal matrix *A* (*m*×*k*). Then the (*m*×*n*) matrix *Y = AX* represents the coordinates of the projected data in the *m*-dimensional (*m* < *k*) space spanned by the rows of *A*. Because the number of possible projections is infinite, it is important to have a technique to pursue a finite sequence of projections that can reveal the most informative structures in the data. Projection pursuit (PP) is a tool that combines both ideas of projection and pursuit. [36] In a typical regression problem, PPR aims to approximate the regression pursuit function *f(x)* by a finite sum of ridge functions with suitable choices of *α_i_* and *g_i_*.
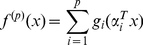
(1)where the *α_i_* values are *m*×*n* orthonormal matrices and *p* is the number of ridge functions. All programs implementing PPR were written in R-script under the R2.9.0 environment [49] and were executed on a Linux-operated Pentium IV with 4Gb of RAM.

### Evaluation of QSAR models

The predictive accuracy of the QSAR models was evaluated in terms of root-mean-square error (RMSE), defined as
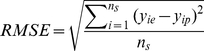
(2)where *n_s_* is the number of known EGFR inhibitory compounds, and *y_ie_* and *y_ip_* are, respectively, the experimental and predicted EGFR inhibitory activities for the *i_th_* compound.

## Results and Discussion

### Best multi-linear regression model

**Figure 2:**
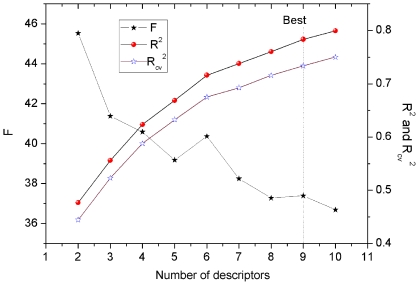
Influence of the number of descriptors on the square of Pearson correlation coefficient (*R^2^*), leave one out (LOO) cross-validation coefficient (*R_cv_^2^*), and *F*-values of the BMLR models.



The best multi-linear regression (BMLR) method was utilized to develop a multi-linear QSAR model and select the most relevant molecular descriptors based on the training set. A variety set of descriptors have been tested for the selection of descriptors in different linear regression models. To avoid model “over-parameterization”, an increase of the squared Pearson correlation coefficient (*R^2^*) by less than 0.02 was chosen as the breakpoint criterion. Fig. 2 shows the number of descriptors *versus* the values of *R^2^*, the leave-one-out (LOO) cross-validation (*R_CV_^2^*), and Fisher's *F*-test. It can be seen that nine descriptors are sufficient to optimize the regression model of the *pIC_50_* of EGFR inhibitors. The optimum model is:

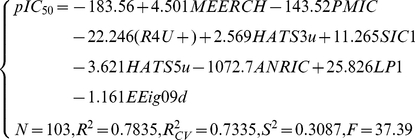
(3)where *N* is the number of compounds in the training set, *R^2^* is the squared Pearson correlation coefficient, *R_CV_^2^* is the squared cross-validation correlation coefficient, *S^2^* is the squared standard deviation, and *F* is the Fisher *F*-test function. There are nine molecular descriptors selected. Three were calculated from CODESSA: *MEERCH* (Min e-e repulsion for a C-H bond), *PMIC* (Principal moment of inertia C), and *ANRIC* (Avg nucleoph. react. index for a C atom); the other five descriptors were obtained from DRAGON: *R4U+*, *HATS3u*, *SIC1*, *HATS5u*, *LP1,* and *EEig09d*.

The descriptor *MEERCH* is a quantum mechanical energy-related descriptor, used to characterize the total energy of the molecule at different energy scales and intramolecular energy distribution using different partitioning schemes. It is calculated as follows:
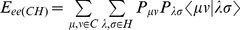
(4)where *P_µv_* and *P_λσ_* are the density matrix elements and 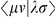 are the electron repulsion integrals on the atomic basis 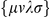. This descriptor refers to the electron repulsion-driven process in the molecule and can be related to the conformational (rotational, inversional) changes or atomic reactivity in the molecule [50]. The descriptor *PMIC* is a geometrical descriptor which related with their 3D-coordinates information of the atoms in the molecule. It is equal to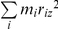, where *m_i_* is the mass of the *i*th atom and *r_iz_* denotes the distance of the *i*th atomic nucleus from the main rotational *z*-axis of the molecule [51]. As an expression of the principal moment of inertia, this descriptor characterizes the distribution of mass in the molecule. The descriptor *ANRIC* is a quantum chemical descriptor of the average value of the atom C nucleophilic (*N_A_*) Fukui reactivity indices [52] for carbon in the molecule, which are defined as follows:
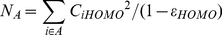
(5)where, *C_iCHOMO_* denotes the *i*th AO coefficient for the highest occupied molecular orbital (HOMO), and *ε_HOMO_* is the energy of these orbitals. The reactivity indices estimate the relative reactivity of carbon in the molecule for the given series of compounds and are related to the activation energy of the corresponding chemical reaction. The descriptor *R4U+*, which belongs to the GETAWAY (GEometry, Topology and Atom-Weights AssemblY) descriptors, is the R maximal autocorrelation coefficient of lag 4/unweighted and R autocorrelation coefficient of lag 5/unweighted, respectively. The *R* and *R+* descriptors are analogously obtained from the leverage/geometry matrix [53]. The descriptors *HATS3u* and *HATS5u* are GETAWAY descriptors defined by the leverage-weighted autocorrelation of lag 3 and lag5/unweighted, respectively. They take into account 3D molecular geometry by using the leverage values as atom weights [53]. The descriptor *SIC1* is one of the information indices of the molecule, the name standing for Structural Information Content index (neighborhood symmetry of 1-order). It is defined by the application of information theory to the chemical and bonding neighborhood of the atoms in the molecule [54]; thus it might reflect molecular polarity and polarizability. *LP1* is the Lovasz–Pelikan index or leading eigen value, and is a topological descriptor. It has been suggested as an index of molecular branching, the smallest values corresponding to chin graphs and the highest to the most branched graphs. In equation (3) the *LP1* contribution has a positive sign, which indicates that the IC_50_ is inversely related to this descriptor; therefore, increasing the branching of molecules leads to a decrease in their IC_50_. The last descriptor, *EEig09d,* is a topological molecular descriptor. It is defined as Eigenvalue 09 from the edge adj. matrix weighted by dipole moments, and encodes the connectivity between graph edges.

**Figure 3:**
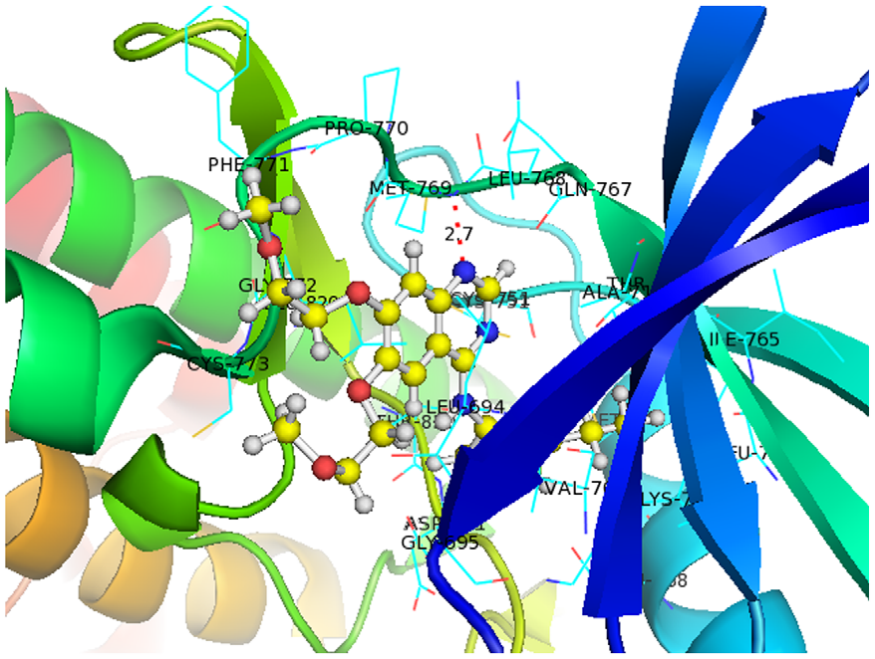
Crystal structure of EGFR bound to the 4-anilinoquinazoline inhibitor Erlotinib (PDB ID: 1M17). EGFR is represented by a cartoon model, with the side chains of the binding site wire-framed in cyan and labeled. The inhibitor is represented by a stick-and-ball model, where carbons are colored in yellow, nitrogens in blue, oxygens in red, and hydrogens in light grey. The hydrogen bond between an amide nitrogen donor of the ligand and the carboxyl group of MET769 in the receptor is plotted as a red dotted line. Figure produced with the PyMOL program [56].

From the above presentation, it is concluded that the selected descriptors can be interpreted reasonably, and the inhibition ability of quinazoline derivatives mainly depends on the following properties: polarizability, activation energy, mass distribution, connectivity, and branching information. In order to facilitate our understanding of the main features of EGFR inhibitors, we also investigated one of the existing X-ray crystal structures of ligand-bound EGFR [55]. As shown in Fig. 3, an amide nitrogen donor of the ligand together with the carboxyl group of MET769 form a hydrogen bond in the EGFR hinge region. Hydrogen-bond interactions play a crucial role in ligand–protein binding; however polarizability was one of the critical factors in forming the interaction in a broader sense, involving both the hydrophilic and hydrophobic regions of the receptor. In fact, the polarizability of the ligand is essential to stabilize any generated hydrogen bonds. Consistently with our QSAR study, Vema et al. [30], who docked the selected drugs to the active binding site of the same EGFR kinase domain, found that the regions of the receptor surface around different branching places exhibited different electronic properties, either electronegative or electropositive. Thus the connectivity and branching information contributed greatly to the docking and interaction of the ligands with EGFR. Such information was helpful to clarify the mechanisms of molecular docking encountered in drug-discovery studies.

**Figure 4:**
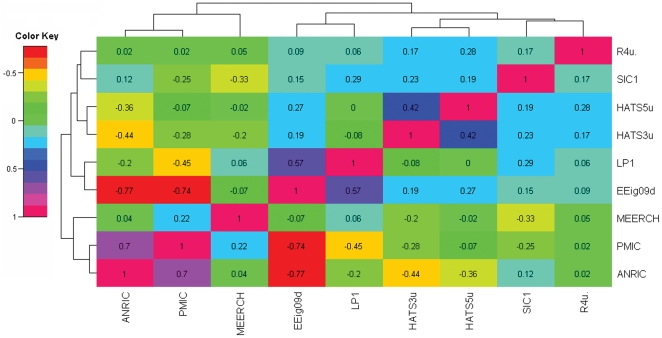
Heat maps of the correlation matrix of the molecular descriptors. Rows and columns are ordered according to a hierarchical clustering (cluster tree lines on the side and top) of the selected molecular descriptors codes.

The correlation matrix of these selected descriptors is shown by a heat map in Fig. 4, produced with the *R*-package gplots [57]. The linear correlation coefficients of all descriptor pairs are at most equal to 0.80 and the majority of them are below 0.3, which demonstrates the relative independence of the selected descriptors. Furthermore, the hierarchical clustering reveals noteworthy contributions of the nature of the different selected molecular descriptors. For example, descriptors *EEig09d* and *LP1*, accounting primarily for the connectivity and branching information of the molecule, cluster together. Similarly, descriptors *HATS3u*, *HATS5u* and *R4u* belong to GETEWAY descriptors, and they are in the same cluster, by representing the information of different atoms in the molecules, and all related with the Cartesian coordinates of the molecule atoms (including hydrogen) in a chosen conformation [58]. Finally, the six descriptors which were calculated from DRAGON were clustered together; and they can be viewed as describing the intrinsic property of the molecules. Descriptors ANRIC and MEERCH are clustered together. They were calculated from quantum chemistry methods, and represent their electron and nucleoph properties in the molecules. They related with the activation energy and electronegativity of the molecule, respectively, and simulated the interactions with other molecules.

**Figure 5:**
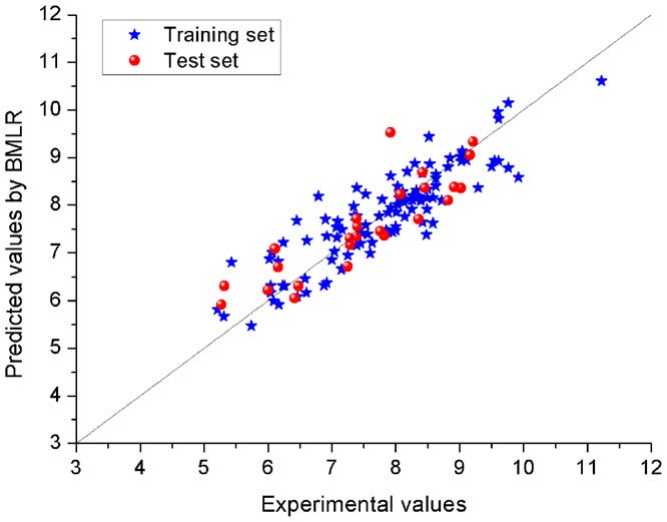
Plot for the training and test EGFR inhibitors of the pIC_50_ values predicted by the BMLR method *versus* the experimental pIC_50_ values.

The predicted pIC_50_ activity values for all the known EGFR inhibitors, including the training and test sets are given in Table S1, where the experimental pIC_50_ values are also listed. Fig. 5 shows the predicted *versus* the experimental pIC_50_ values for the training and test sets, respectively. There is an obvious correlation between the predicted and experimental values of pIC_50_, with the square of Pearson correlation coefficients 0.7835 and 0.7595 for the training and test sets, respectively. The whole statistical parameters of the BMLR model are given in Table 1. There is no notable difference in the correlation coefficient for the test and training data, confirming that the model was indeed not “over-trained”.

**Table 1:** Comparison of the statistical results between the BMLR and GS-PPR models.

Parameter	Sets	BMLR	GS-PPR	Ref. [30]
R^2^	Training set	0.7835	0.8534	0.8492
	Test set	0.7595	0.8116	0.5325
	All set	0.7808	0.8461	0.7691
RMSE	Training set	0.5656	0.4345	0.4347
	Test set	0.5280	0.5040	0.8455
	All set	0.5355	0.4489	0.5514

**Note**: *BMLR*: best multi-linear regression; GS-*PPR*: grid search-projection pursuit regression. *R^2^*: squared Pearson correlation coefficient between the experimental and predicted pIC_50_ values. *RMSE*: root-mean-square error between the experimental and predicted pIC_50_ values.

### Principal component analysis of the selected descriptors

**Figure 6:**
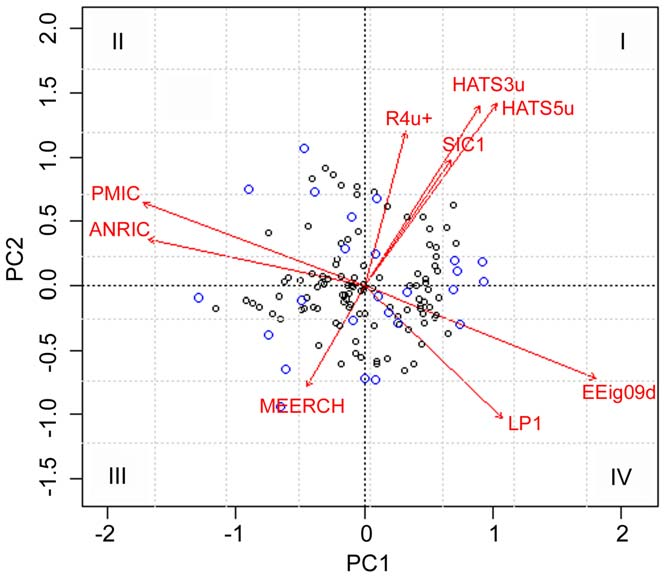
Principal component analysis of the nine selected descriptors. Arrows indicate the directions of the variable vectors in the principal component space. Black circles denote compounds from the training set, blue circles those from the test set.

Principal component analysis (PCA) is always used to reduce the dimensionality of multidimensional variables and analyze complex intrinsic features among variables. In the current research, PCA method was performed using the selected nine descriptors with the aim to show the spatial location of every drug, and also check the distribution of the drugs in the training and test sets. The two major principal components were given here. The explained variance of these two components is 50.38% of the total information (PC1 explained variance equals to 33.98%), thus the most useful information was interpreted in the first two PCs. Fig. 6 illustrates a loading plot of these two components for the training and test sets. From this figure, it can be seen that the samples in the both training and test sets are well balanced and evenly scattered over the whole space occupied by dissimilar plotting symbols. This confirmed that the drugs in the training set can be used as the representative samples of the whole data set, and the splitting method is also reliable for the assessment of the predictive ability and performance of different models.

Furthermore, it also illustrates that the nine selected variables have various directions and various sizes in this figure. These descriptors clearly distributed in the four different regions of rectangular coordinate system: R4u+, HATS3u, HATS5u and SIC1 in quadrant (I); RMIC and ANRIC in quadrant (II), MEERCH in quadrant (III), and EEig09d and LP1 in the last quadrant (IV) . All these information explored here consisted with the results of former cluster analysis.

### Projection pursuit regression model

**Figure 7:**
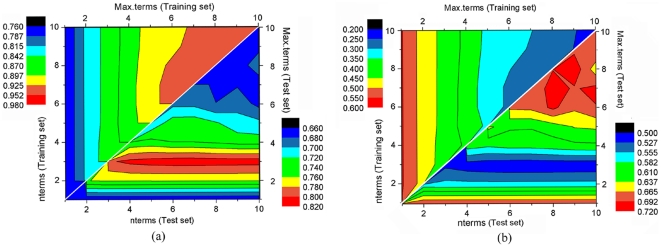
Statistical parameters of the training set and the test set during the optimization process of the PPR parameters by the grid search method. (a): *R^2^*, (b): RMSE.

After building the linear BMLR model, projection pursuit regression (PPR) was applied to effectively project the nine descriptors to a lower-dimensional space, and to perform a non-linear regression in this lower dimensional space; the goal of the regression was to correlate the EGFR inhibitory activity with the structural information. The PPR approach requires the optimization of several parameters, including ‘nterms’ and ‘max.terms’, which represent the number of ridge terms included in the final model and the maximum number of ridge terms for building the model, respectively; the parameter ‘df’ defines the smoothness of each ridge term by the requested equivalent degrees of freedom; the levels of optimization (parameter ‘optlevel’) differ in how thoroughly the models are refitted. At level 0 the existing ridge terms are not refitted. At level 1 the projection directions are not refitted, but the ridge functions and the regression coefficients are. Levels 2 and 3 refit all of the terms and are equivalent for one response; level 3 is more careful to re-balance the contributions from each regression at each step and so is slightly less likely to converge to a saddle point of the sum of squares criterion. Since the traditional PPR method usually adopts a single-fact correction analysis, the models they produce tend to be only local optima. Here, the grid-search (GS) method was employed, which relies instead on multi-fact correction analysis, thereby producing a final model that is generally closer to the global optimum [59]. The results indicate that the ‘optlevel’ and ‘df’ parameters influenced the optimization only slightly. Figures 7(a) and 7(b) show the values of *R^2^* and *RMSE*, respectively, as a function of ‘max.terms’ and ‘nterms’, the two most important optimization parameters. The optimum values of ‘nterms’, ‘max.terms’, ‘df’, and ‘optlevel’ are determined as 3, 7, 8, and 1, respectively. In order to assess the internal predictability of the training set, a 5-fold cross validation is typically used, providing an estimate for the mean performance of a model. The values of the statistical parameters of the 5-fold cross validation were *R_cv_^2^* = 0.7709, and *RMSE* = 0.6186. Furthermore, the results of the test set prediction were confirmed by the external prediction of the regression model. These data suggest that the model we proposed has a robust prediction power.

**Figure 8:**
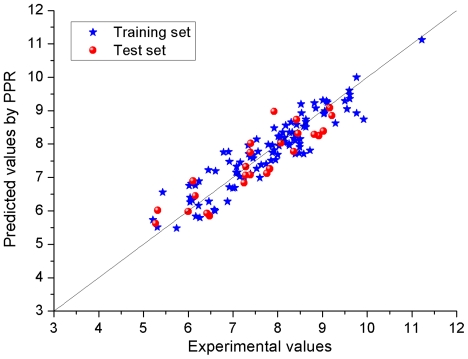
Plot for the training and test EGFR inhibitors of the pIC_50_ values predicted by the GS–PPR method *versus* the experimental pIC_50_ values.

The predicted results and the statistical parameters of the optimal PPR model are shown in Tables S1 and 1, respectively. The scatter plot of the predicted *versus* the experimental pIC_50_ values is given in Fig. 8. From Fig. 8 and Table S1 it can be seen that the predicted values are in good agreement with the experimental values for almost all the compounds.

### Comparison of results of obtained by different models

In order to check the superiority of these two different models (BMLR and GS-PPR), the predicted results and the statistical parameters for the training and the test sets were collected together and shown in Tables S1 and 1, separately. As it can be seen from this table, the improved non-linear regression method GS-PPR outperforms the BMLR model according to the *R* ^2^ and *RMSE*, and it shows much better predictive ability, and its corresponding predicted results indicate an appropriate fit of the model. Previously, Vema et al. [30] used the 3D-QSAR method molecular field analysis (MFA) and receptor surface analysis (RSA) to investigate the inhibitory activities of the same data set. The squared Pearson correlation coefficient (*R^2^*) of their best model (RSA) is 0.8492 for the training set and 0.5325 for the test set. All of the other statistical parameters were collected in Table 1. By comparing these results, it can be concluded that the GS-PPR method is a simple but with powerful predictive capability tool as to the inhibitory activity of potential anti-EGFR drugs.

### Conclusions

We have explored the features of potential inhibitors of epidermal growth factor receptor, a vital protein target involved in clinical anticancer therapies, based on linear and non-linear QSAR models. A new non-linear QSAR method for the prediction of EGFR inhibitory activity was developed, which combines the grid search (GS) and projection pursuit regression (PPR) techniques to infer biological activity from a set of molecular descriptors; these were selected by the best multi-linear regression (BMLR) exclusively from structural information. The GS-PPR model showed a better predictive ability than the traditional linear QSAR model, demonstrating that the combination of PPR and GS is a valuable strategy for QSAR model building, at least for the prediction of EGFR inhibitors. In addition, this investigation shows that the structural features of quinazoline derivatives are most relevant to quinazoline derivatives inhibition — namely, polarizability, activation energy, mass distribution, connectivity, and branching. The set of EGFR inhibitors, real or hypothetical, which can possibly be examined by such studies, is large and heterogeneous, due to the purely structural nature of the molecular descriptors. The approach can be easily extended to other cheminformatic and bioinformatics investigations, since the small number of parameters to be optimized makes the training procedure generally simple.

## Supporting Information

Table S1 Structures and EGFR inhibitory activities of 128 known EGFR inhibitors.
(PDF)
