# Supplementary material for: Prediction of Inhibitory Activity of Epidermal Growth Factor Receptor Inhibitors Using Grid Search-Projection Pursuit Regression Method
Source: PLoS One. 2011 Jul 21;6(7):e22367. doi: 10.1371/journal.pone.0022367 (PMC3141047; doi:10.1371/journal.pone.0022367)
Supplement: Table S1 — Structures and EGFR inhibitory activities of 128 known EGFR inhibitors. (PDF) [file pone.0022367.s001.pdf]

Table S1. Structures and EGFR inhibitory activities of 128 known EGFR inhibitors.

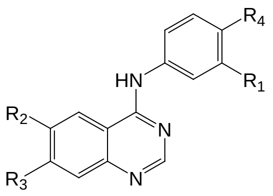

A

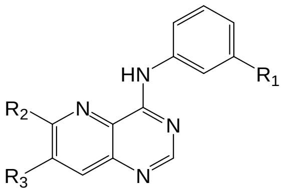

B

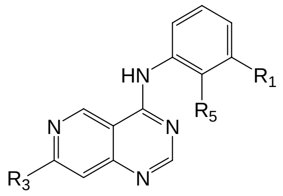

C

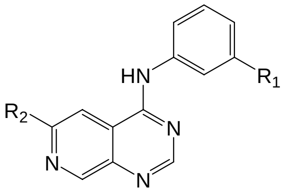

D

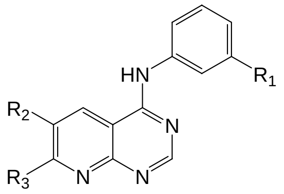

E

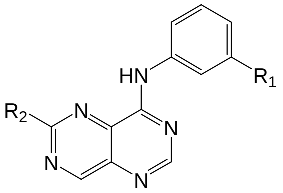

F

No.	Class	R_1	R_2	R_3	R_4	R_5	pIC_{50}	BMLR	GS-PPR
1	A	-	-	-	-	-	6.46	6.09	6.28
2	A	Me	-	-	-	-	6.04	6.32	6.26
3	A	Cl	-	-	-	-	7.63	7.21	7.38
4	A	Br	-	-	-	-	7.56	7.39	7.77
5	A	I	-	-	-	-	7.09	7.67	7.15
6	A	CF_3	-	-	-	-	6.23	6.32	6.15
7	A	Br	NO_2	-	-	-	6.04	6.17	6.40
8	A	Br	OMe	-	-	-	6.45	7.68	7.23
9*	A	Br	-	NO_2	-	-	6.00	6.22	5.98
10	A	Br	-	OMe	-	-	8.00	7.47	7.68
11	A	Br	OH	OH	-	-	9.76	8.78	8.92
12	A	Br	NH_2	NH_2	-	-	9.92	8.58	8.74
13	A	F	-	-	-	-	7.25	6.94	7.01
14*	A	-	OMe	-	-	-	7.25	6.71	6.84
15*	A	-	NH_2	-	-	-	6.11	7.08	6.90
16	A	CF_3	NH_2	-	-	-	6.24	7.22	6.89
17	A	-	OMe	-	-	-	6.92	6.38	6.71
18	A	-	-	NH_2	-	-	7.00	6.85	6.68
19	A	CF_3	-	NH_2	-	-	8.84	7.38	7.73
20	A	F	-	NO_2	-	-	5.21	5.82	5.73
21	A	Cl	-	NO_2	-	-	6.09	5.99	6.32
22	A	I	-	NO_2	-	-	6.26	6.31	5.80
23	A	-	OMe	OMe	-	-	7.53	8.23	8.14
24	A	F	OMe	OMe	-	-	8.42	8.68	8.58
25	A	Cl	OMe	OMe	-	-	9.50	8.81	9.29
26	A	I	OMe	OMe	-	-	9.05	9.08	8.99
27	A	CF3	OMe	OMe	-	-	9.61	8.93	9.35
28	A	Br	NHMe	-	-	-	8.39	8.10	7.71

29	A	Br	$N(Me)_2$	-	-	-	7.07	7.54	7.16
30	A	Br	NHCOOMe	-	-	-	7.92	7.95	7.81
31	A	Br	-	OH	-	-	8.32	8.31	8.35
32	A	Br	-	NHAc	-	-	7.39	8.36	7.74
33	A	Br	-	NHMe	-	-	8.15	8.12	8.20
34*	A	Br	-	NHEt	-	-	7.92	9.53	8.98
35	A	Br	-	$N(Me)_2$	-	-	7.95	7.43	7.53
36*	A	Br	NH_2	NHMe	-	-	9.16	9.06	9.09
37	A	Br	NH_2	$N(Me)_2$	-	-	6.79	8.19	7.76
38*	A	Br	NH_2	OMe	-	-	8.42	8.69	8.73
39	A	Br	NH_2	Cl	-	-	8.18	8.70	8.56
40	A	Br	NO_2	NHMe	-	-	7.16	7.49	7.46
41*	A	Br	NO_2	OMe	-	-	7.82	7.36	7.26
42	A	Br	NO_2	Cl	-	-	7.60	6.99	6.99
43	A	Br	OEt	OEt	-	-	11.22	10.61	11.12
44	A	Br	OPr	OPr	-	-	9.76	10.15	10.00
45	A	H	OMe	OMe	Br	-	9.01	9.02	9.31
46	B	Br	-	-	-	-	7.46	7.41	7.67
47	B	Br	NH2	-	-	-	8.11	8.28	8.35
48	B	Br	Cl	-	-	-	7.74	7.77	7.40
49	B	Br	F	-	-	-	7.35	7.78	7.71
50	B	Br	NHMe	-	-	-	8.50	8.34	8.15
51	B	Br	$N(Me)_2$	-	-	-	8.01	7.85	8.23
52*	B	Br	OMe	-	-	-	8.36	7.70	7.78
53	C	Br	-	-	-	-	7.45	7.19	7.59
54	C	Br	-	NHAc	-	-	7.53	7.58	7.27
55	C	Br	-	F	-	-	7.88	7.42	7.96
56*	C	Br	-	OMe	-	-	7.40	7.55	8.02
57	C	-	-	NH_2	-	-	6.60	6.17	6.01
58*	C	NO_2	-	NH_2	-	-	7.39	7.34	7.08
59	C	-	-	NH_2	-	Br	6.61	7.26	7.20
60	C	Br	-	NH_2	-	-	8.00	7.55	7.69
61	C	-	-	NH_2	Br	-	7.04	7.02	6.69
62*	C	-	-	NH_2	CF_3	-	5.32	6.31	6.02
63	C	-	-	NH_2	-	OMe	5.43	6.80	6.56
64	C	OMe	-	NH_2	-	-	6.88	6.32	6.28
65	C	-	-	NH_2	OMe	-	6.17	5.92	5.83
66*	C	-	-	NH_2	-	NH_2	5.27	5.92	5.62
67	C	$N(Me)_2$	-	NH_2	-	-	5.74	5.47	5.49
68	C	-	-	NH_2	$N(Me)_2$	-	5.31	5.67	5.51
69	C	F	-	NH_2	-	-	6.07	7.00	6.82
70	C	Cl	-	NH_2	-	-	6.92	7.35	7.48
71	C	OH	-	NH_2	-	-	7.15	6.65	7.03
72	C	Me	-	NH_2	-	-	7.39	7.15	7.11

73*	D	Br	-	-	-	-	7.29	7.16	7.32
74*	D	Br	Cl	-	-	-	7.39	7.71	7.75
75	D	Br	F	-	-	-	6.90	7.70	7.77
76	D	Br	OMe	-	-	-	8.58	7.62	7.71
77	D	Br	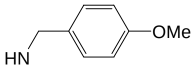	-	-	-	8.63	8.64	8.74
78	E	Br	-	-	-	-	6.16	6.82	6.76
79	E	Br	-	NH_2	-	-	6.02	6.87	6.76
80*	E	Br	-	F	-	-	6.16	6.70	6.45
81*	E	Br	-	NHMe	-	-	7.28	7.30	7.06
82*	E	Br	-	$N(Me)_2$	-	-	6.48	6.31	5.85
83	E	Br	-	OMe	-	-	6.58	6.46	6.04
84	F	H	NHMe	-	-	-	7.88	7.84	7.75
85	F	Br	Cl	-	-	-	7.08	7.32	7.32
86	F	Br	NH_2	-	-	-	8.82	8.81	9.23
87	F	Br	NHMe	-	-	-	9.11	8.94	9.26
88*	F	Br	$N(Me)_2$	-	-	-	9.02	8.36	8.39
89	F	Br	OMe	-	-	-	8.42	7.65	8.24
90	F	Br	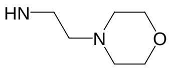	-	-	-	9.09	8.98	9.29
91	F	Br	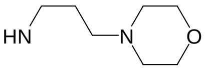	-	-	-	8.53	8.86	8.92
92	F	Br	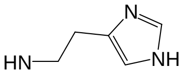	-	-	-	9.60	9.96	9.61
93	F	Br	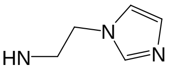	-	-	-	8.63	8.57	8.51
94*	F	Me	Cl	-	-	-	6.42	6.05	5.92
95*	F	Me	NH_2	-	-	-	7.76	7.45	7.12
96	F	Me	NHMe	-	-	-	8.36	8.17	8.16
97	F	Me	$N(Me)_2$	-	-	-	8.39	8.13	8.10
98	F	Me	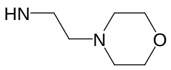	-	-	-	8.63	8.40	8.66
99	F	Me	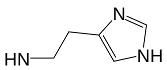	-	-	-	8.52	9.43	9.19
100	C	Br	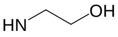	-	-	-	9.61	9.82	9.48
101	C	Br	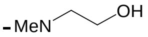	-	-	-	8.58	8.16	8.53
102	C	Br	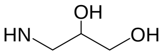	-	-	-	9.03	8.92	8.90
103	C	Br	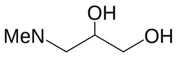	-	-	-	8.49	7.92	7.91
104	C	Br	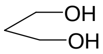	-	-	-	7.85	7.48	7.51
105	C	Br	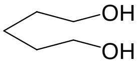	-	-	-	7.92	8.61	8.48

106	C	Br	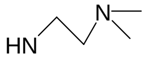	-	-	-	7.34	7.98	7.96
107	C	Br	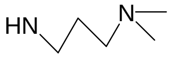	-	-	-	8.05	8.15	8.19
108	C	Br	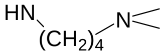	-	-	-	8.13	8.06	8.05
109*	C	Br	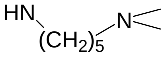	-	-	-	8.07	8.23	8.03
110	C	Br	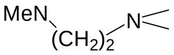	-	-	-	7.39	7.77	7.61
111	C	Br	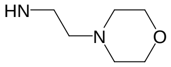	-	-	-	8.49	8.16	8.01
112	C	Br	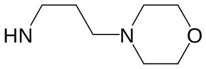	-	-	-	8.72	8.10	7.81
113	C	Br	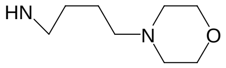	-	-	-	8.26	8.19	7.96
114	C	Br	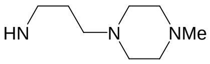	-	-	-	8.30	8.88	8.65
115	C	Br	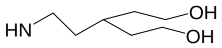	-	-	-	8.03	8.39	8.24
116*	C	Br	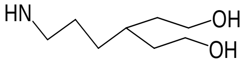	-	-	-	8.92	8.38	8.25
117	C	Br	$NHNH_2$	-	-	-	8.14	7.76	8.07
118	C	Br	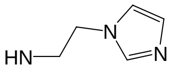	-	-	-	9.29	8.37	8.63
119	C	Br	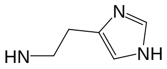	-	-	-	9.04	9.13	8.93
120*	C	Br	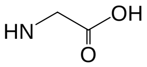	-	-	-	8.82	8.10	8.29
121*	C	Br	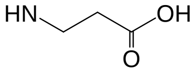	-	-	-	9.21	9.33	8.85
122	C	Br	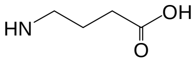	-	-	-	9.55	8.94	9.05
123	C	Br	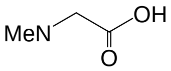	-	-	-	7.79	8.12	7.98
124	C	Me	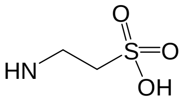	-	-	-	8.85	8.99	9.07
125	C	Me	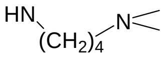	-	-	-	8.26	8.10	8.35
126	C	Me	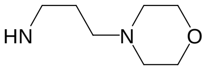	-	-	-	8.03	8.01	7.95
127	C	Me	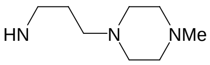	-	-	-	8.25	7.91	8.02

128*	C	Me	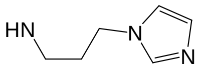	-	-	-	8.45	8.36	8.32

Note: *: test set; BMLR: Best multi-linear regression; GS-PPR: Grid-search – projection pursuit regression.
